# Application of a stoichiometric bioenergetic approach and whole-body protein synthesis to the nutritional assessment of juvenile *Thenus australiensis*

**DOI:** 10.1038/s41598-023-41070-z

**Published:** 2023-09-01

**Authors:** Andrea Williamson, Chris G. Carter, M. Basseer Codabaccus, Quinn P. Fitzgibbon, Gregory G. Smith

**Affiliations:** Institute for Marine and Antarctic Studies (IMAS), University of Tasmania, Private Bag 49, Hobart, TAS 7001 Australia

**Keywords:** Metabolism, Animal physiology, Respiration

## Abstract

The present study successfully combined a stoichiometric bioenergetic approach with an endpoint stochastic model to simultaneously determine specific dynamic action, metabolic substrate use and whole-body protein synthesis in juvenile slipper lobster *Thenus australiensis*. Juvenile lobsters were fasted for 48 h to investigate routine metabolism before receiving a single meal of formulated feed containing 1% ^15^N-labeled Spirulina. Postprandial oxygen consumption rate, dissolved inorganic carbon, and total nitrogen excretion returned to the pre-feeding level within 24 h. The rate of whole-body protein synthesis was 0.76 ± 0.15 mg CP g^−1^ day^−1^, with a significant reduction from 24 to 48 h post-feeding. The postprandial increase in whole-body protein synthesis accounted for 13–19% of total oxygen uptake. Protein was the primary energy substrate for 48 h fasted (45% oxygen consumption) and post-feeding lobster (44%), suggesting that dietary protein was not efficiently used for growth. The secondary energy substrate differed between carbohydrates in 48 h fasted and lipids in post-feeding lobsters. The present study recommends integrating protein synthesis into protein requirement experiments of marine ectotherms to acquire a more comprehensive picture of protein and energy metabolism and nutritional physiology crucial for formulating cost-effective aquafeeds.

## Introduction

The slipper lobster, *Thenus australiensis*, has become a research focus due to its commercial value and as a target species for intensive aquaculture^1–3^. The recent closure of the *T. australiensis* life cycle in captivity using only formulated feeds^3^ has laid the foundation for sustainable aquaculture development. A critical step for developing commercial aquaculture for the species is to improve our understanding of nutritional physiology, specifically protein metabolism, to support cost-effective formulated feed development. There is substantial information about the nutrient requirements and physiology of spiny lobsters^4–7^. However, information on the nutritional physiology of *T. australiensis* is limited^2,8,9^, although it is well known that in the wild, slipper lobsters are primarily carnivorous scavengers preferring small benthic invertebrates including molluscs, polychaetes and crustaceans^10^. To our knowledge, information on nitrogen flux and protein turnover is lacking, both of which are vital for successful aquafeed development.

Protein turnover can be divided into constituent processes, protein synthesis, protein growth and protein degradation^11^. Protein growth occurs when whole-body protein synthesis (WBPS) exceeds protein degradation^12,13^. Protein synthesis is directly related to the quantity and quality (amino acid profile) of ingested proteins. When crustaceans are supplied with amino acids above the optimum relative amino acid ratio, they are deaminated and excreted in a range of nitrogenous compounds, predominantly as ammonia-N and, to a lesser extent, urea-N and amino acids^13,14^. Hence, measuring WBPS and protein turnover provides an approach to assess imbalances and deficiencies in dietary amino acids and energy and can help close knowledge gaps in crustacean nutrition^13,15^. However, up to now, research on protein turnover and protein synthesis in aquatic ectotherms has focused on fish and less on crustaceans^6,16,17^. As with other animals, protein synthesis is a major energy-demanding process in crustaceans, and previous studies suggested that protein synthesis rates and efficiencies are species-specific and affected by development stages^18,19^. Overall, crustaceans displayed higher protein retention efficiency (> 80%) compared to fish (32–69%)^15,18,19^.

Previous studies identified protein synthesis as an essential contributor to the specific dynamic action (SDA) in crustaceans, as evidenced by the proportional increase in WBPS and oxygen consumption rates (ṀO_2_) following a meal^20^. Specific dynamic action is the postprandial increment in metabolism, representing energetic costs from ingestion, digestion, absorption and metabolic processing of energy substrates^16,21^. It reflects the balance of available nutrients and mainly represents post-absorptive metabolic costs^13,16^. The WBPS in shore crabs *Carcinus maenas* accounted for 20–37% (15 °C) of the postprandial increase in ṀO_2_^20^, while in the isopod *Glyptonotus antarcticus*, WBPS accounted for 68–78% (0 °C)^22^.

Three methods are used to measure in vivo protein synthesis: constant infusion, flooding dose and stochastic endpoint^12,19^. However, most studies on crustaceans have used a flooding dose of a radioactive amino acid (^3^H-phenylalanine)^15^. A novel non-destructive approach combines a stoichiometric bioenergetic method with an endpoint stochastic model, allowing the simultaneous determination of the metabolic substrate use, SDA and WBPS^6^. This combined approach offers potential as it can examine the balance between metabolic energy substrate use on an aquatic ectotherm at any time and provides precise measurements of metabolic energy substrate use under different feeding conditions. The stoichiometric bioenergetic approach is based on the determination of respiratory and nitrogen quotient, derived from the simultaneous measurement of respiratory gas (O_2_ and CO_2_) exchange and nitrogenous (ammonia and urea) excretion^23^. This approach has been successfully applied to spiny lobster *Sagmariasus verreauxi* to determine the relationship between WBPS and dietary protein levels^6^.

The investigation of WBPS is the key to understanding daily protein-nitrogen flux in aquatic ectotherms, providing critical information for optimising dietary protein to formulate cost-effective aquafeeds^24,25^. Consequently, the present study aimed to establish baseline information about SDA, metabolic substrate use, and WBPS in juvenile *T. australiensis* using a stoichiometric bioenergetic approach and an endpoint stochastic model. Since ^15^N labelled Spirulina was used to determine WBPS, an adjunct experiment was conducted to evaluate the effect of Spirulina levels on the apparent digestibility (AD) of the experimental feed and Spirulina itself.

## Methods and material

### Feed manufacture

Feeds were made for two experiments, and manufacture was as per Wirtz, et al.^2^. For the apparent digestibility experiment, Spirulina powder was incorporated into a commercial in confidence basal mix including 0.1% of the inert marker yttrium oxide at an inclusion rate of 0%, 15% and 30% to produce three experimental feeds (Ref, SP1 and SP2, Table 1). To determine protein synthesis, ^15^N labelled Spirulina was incorporated at an inclusion rate of 1% into a commercial in confidence basal mix, including 0.1% of the inert marker yttrium oxide. After mixing the feed ingredients with water, the resultant dough was cold extruded into 1.5 mm feed strands using a pasta extruder (La Monferrina Dolly II). Freshly extruded feed strands were set for 12 h at 4 °C^26^. After setting, the feed strands were cut into 10 mm length pellets and stored in a fridge. Fresh feeds were made fortnightly, and a subsample of these fortnightly batches (4.02 ± 0.02 g fresh weight) was stored at − 20 °C for chemical analysis (Table 1).

**Table 1:** Experimental feed formulation and chemical composition on a dry matter basis.

Ingredient	Digestibility experiment			Protein synthesis
	Ref	SP1	SP2	PS
Ingredients (g Kg^−1^)				
Basal mix^A^	999	699	849	989
Yttrium oxide	1	1	1	1
Spirulina powder^B^	0	300	150	10^C^
Total	1000	1000	1000	1000
Chemical composition (g kg^−1^)				
Dry Matter	944.7	924.0	915.0	914.1
Crude Protein	565.0	619.0	632.2	588.5
Total Lipid	70.9	73.2	85.2	71.4
Ash	164.9	153.0	140.2	162.5
NFE^D^	199.2	15.5	14.2	177.6
Gross Energy (MJ Kg^-1^)^E^	19.8	20.4	21.0	20.0

Experimental feeds were subsampled (*n* = 2 per feed) to reflect the average feed composition over the experimental phase.

^A^Commercial in confidence basal mix.

^B^Spirulina powder, paddymelon, The Melbourne Food Depot.

^C^Algal lyophilised cells-^15^N (Spirulina), 98 atom % ^15^N, Sigma-Aldrich.

^D^NFE (Nitrogen free extract) = 100 − (crude protein + total lipid + ash).

^E^Calculated by using factors 23.9 MJ kg^−1^, 39.8 MJ kg^−1^ and 17.6 MJ kg^−1^ for proteins, lipids and carbohydrates (NFE), respectively^27^.

### Experimental animals

Juvenile *T. australiensis* were hatchery-reared from egg by a commercial lobster hatchery (Ornatas, Toomulla Beach, Australia). After arrival, lobsters were maintained in experimental aquaria (0.38 m length × 0.24 m width × 0.25 m height, 18 L) at the Institute for Marine and Antarctic Studies (IMAS), Hobart, Australia.

### Apparent digestibility of Spirulina

A total of 57 juvenile lobsters with an average (± S.E.) initial wet weight of 26.0 ± 1.4 g were randomly allocated to 9 experimental tanks (038 m length × 0.24 m width × 0.25 m height, 18 L). Three tanks were randomly assigned to each of the three experimental feed treatments. Tanks were supplied with filtered, ozonated seawater at a rate of six exchanges h^−1^ and maintained under a 12:12 h blue light: dark photoperiod, set up as a flow-through culture system. Dissolved oxygen (104.2 ± 0.7% saturation), salinity (33.8 ± 0.0 ppt), pH (8.2 ± 0.0) and temperature (26.6 ± 0.7 °C) were recorded daily to ensure high water quality. Experimental feeds were supplied in excess of requirements continuously over 18 h d^−1^ (approximately 15:00 to 09:00 h daily) using belt feeders, with a daily ration of approximately 2% of body weight on a wet weight: wet weight basis. Belt feeders were loaded daily with a pre-weighed ration of feed derived from each tank's initial total lobster biomass. The feed ration was adjusted through the assessment of mortalities. Uneaten feed was cleaned by siphon. Mortalities and moulting events were recorded daily, and exuviae were removed from the tanks as soon as they were observed to prevent animals from feeding on them.

Apparent digestibility was measured as described in Wirtz, et al.^2^. Briefly, after six days of acclimatisation to the assigned experimental feeds, faeces were collected daily from each tub following the first feeding at 15:00. Faeces were collected with a disposable pipette onto a 250 µm screen and rinsed with distilled water to remove the salt. Daily faecal collection from a single tank was pooled over 48 days and stored at − 20 °C until chemical analyses. The apparent digestibility coefficient (ADC) of the reference and the experimental feeds was calculated according to^2,28^:

$$ADC_{DM} \left( \% \right) = \left( {1 {-} Y_{Feed} /Y_{Faeces} } \right) \times 100$$

$$ADC_{N} \left( \% \right) = \left[ {1 - \left( {Y_{Feed} /Y_{Faeces} } \right) \times \left( {\% N_{Faeces} /\% N_{Feed} } \right)} \right] \times 100 ,$$

where ADC_DM_ represents the ADC of dry matter (DM) in the feed; Y_Feed_ and Y_Faeces_ signify the proportion of the inert marker yttrium oxide in the feed and faeces, respectively; ADC_N_ represents the ADC of nutrients crude protein (CP), total lipid (TL) and gross energy (GE); N_Feed_ and N_Faeces_ are proportion (%) of CP, TL and GE in the feed and faeces, respectively.

The apparent digestibility of Spirulina was calculated based on the 70:30 and 85:15 ratio of reference feed to test feed^29^:

$$AD_{Ing} \left( \% \right) = AD_{TF} + \left( {AD_{TF} - AD_{RF} ) \times (P_{RF} \times N_{RF} } \right)/\left( {P_{Ing} \times N_{Ing} } \right),$$

where AD_Ing_% = AD of nutrients in Spirulina; AD_TF_ = AD of nutrients in the test feed; AD_RF_ = AD of reference feed; P_RF_ = basal mix proportion; P_Ing_ = Spirulina proportion; N_RF_ = nutrient concentration in reference feed and N_Ing _= nutrient concentration in Spirulina.

### Oxygen consumption and protein synthesis

#### Experimental setup

After the digestibility experiment, the remaining 38 lobsters were transferred from the experimental tanks into individual cylindrical vessels (3 L) for holding during acclimatisation to constant dim light to avoid interference from circadian rhythms^17^. During acclimatisation, lobsters were fed ad libitum with the reference feed of the digestibility experiment but formulated with 1% unlabelled Spirulina. After four weeks of acclimatisation, lobsters were closely monitored for moulting events. Five lobsters with an average weight of 53 ± 2 g wet weight (WW, 9.62 ± 0.32 g dry weight (DW)) were used for the experiment. As soon as lobsters showed a constant feed intake (about 1% of their body weight) post-moult, they were weighed and transferred to the cylindrical experimental vessels (3 L) that were equipped with a submersible aquarium pump (AP210 Water feature pump, AQUAPRO, Forrestdale, Australia), luminescent dissolved oxygen optode (Hach LDO, HQ40d, Hach Company, USA) and an air stone connected to the central air supply. The seawater level was maintained at 2.8 L + body volume. Lobsters were fasted for 48 h, ensuring the same post-absorptive status.

#### Oxygen consumption rate and SDA

The oxygen consumption rate (ṀO_2_, mg O_2_ g DW^−1^ h^−1^) was determined according to Wang, et al.^6^. During the last 2 h of the 48 h fasting period, the routine metabolic rate (RMR, mg O_2_ g^−1^ h^−1^) of each lobster was determined (from 06:00) based on the measurement of ṀO_2_^30,31^. Briefly, ṀO_2_ was calculated from the decline in dissolved oxygen concentrations in the experimental vessel during the experiment measured with a luminescent dissolved oxygen optode (Hach LDO, HQ40d, Hach Company, USA). Experimental vessels were covered with a blue transparent solar pool cover (Intex Development Co., Ltd., Hong Kong) to limit oxygen diffusion with the environment. Aeration and seawater flow were manually halted for a 20 min ṀO_2_ measurement period, followed by a 10 min re-oxygenation period. The halt-restart process was repeated three times, and the RMR was determined as the mean of the three ṀO_2_ measurements, where the background ṀO_2_ for each lobster was subtracted^32–34^. Background ṀO_2_ was determined 2 h before stocking the animal by the same process but in an experimental vessel without a lobster.

The RMR measurements were followed by a 1.5 h feeding period. Lobsters were fed with ^15^N labelled experimental feed at a ration of 1.5% of their body weight (average body weight 0.71 ± 0.03 g DW). Subsequently, uneaten feed was collected for apparent feed intake (AFI) determination. The seawater level in the experimental vessel was dropped to 1.3 L + body volume by siphoning 1.5 L of seawater into a 5 L plastic flask via Tygon E-3603 tubing (Saint-Gobain Performance Plastics, Charny, France). After that, 1.5 L of freshly filtered seawater was added into the experimental vessel, and the lobster was subjected to the 20 min seawater addition and ṀO_2_ measurement protocol (as described above) at 2 h intervals for the first 12 h, followed by a 24 h and 48 h to provide nine postprandial ṀO_2_ measurements for the determination of SDA. Six SDA variables were individually identified: (1) SDA_peak_, determined as the peak post-feeding ṀO_2_ (mg O_2_ g DW^−1^ h^−1^); (2) time to SDA_peak_, determined as the time from feeding to SDA_peak_ (h); (3) SDA duration, determined as the duration that the average post-feeding ṀO_2_ remained greater than the RMR (h); (4) SDA magnitude (TṀO_2_, mg O_2_ g DW^−1^), calculated by the total increase in ṀO_2_ above the RMR; (5) E_SDA_ (J g^−1^) was determined using a simplified traditional approach where SDA magnitude was converted to energy using an empirical oxycalorific coefficient (Q_OX_) of 13.84 J mg^−1^^35^ and using a stoichiometric bioenergetic approach^17^, where E_SDA_ = 11 × TṀO_2_ (mg O_2_ g DW^−1^) + 2.6 × T*M*CO_2_ (mg g DW^−1^) − 9.5 × T*M*TAN (mg g DW^−1^) − 2.44 × T*M*urea (mg g DW^−1^), representing the accumulated CO_2_, NH_3_ and urea excretion during SDA, respectively; (6) SDA coefficient (C_SDA_, %) calculated by dividing E_SDA_ by the energy in the ingested feed (J g^−1^).

#### Seawater sampling

During the RMR determination, a 20 mL seawater sample was collected within 3 s at the start and end of each ṀO_2_ measuring period via a 20 mL syringe (Terumo Co., Ltd., Japan) to determine routine excretion rates of total dissolved inorganic carbon (*M*DIC, μmol g DW^−1^ h^−1^), total ammonia-N (*M*TAN, mg g DW^−1^ h^−1^) and urea-N (*M*urea-N, µmol g DW^−1^ h^−1^), after correction for background levels. The methods used for seawater sampling were based on Wang, et al.^17^. In brief, 14 mL seawater from the collected 20 mL was dispensed into a 12 mL precooled glass vial (Labco Limited, Lampeter, UK) until overflow to minimise air‐water gas exchange and disinfected with 3.6 μL of saturated mercuric chloride. The dispensation and disinfection were completed within 1 min. The vial was subsequently capped and stored at 4 °C until *M*DIC measurement. The remaining 6 mL were used to determine nitrogenous excretion, with 3 mL sealed in a 10 mL disposable plastic vial for *M*TAN measurement, disinfected with 10% chloroform to prevent bacterial activity and the remaining 3 mL sealed in another 10 mL vial for *M*urea-N measurements. Seawater samples for *M*TAN and *M*urea-N measurement were stored at − 20 °C within 20 min after sampling and thawed at room temperature before analysis.

After feeding, the siphon-collected 1.5 L seawater sample was immediately acidified with 3.75 mL of 4 M HCl and transferred and stored at 4 °C in a 5 L round bottom glass flask (Schott Duran, Mainz, Germany) for the determination of the initial ^15^N concentration^36,37^. Concurrent with postprandial ṀO_2 _measurements, 20 mL seawater samples were collected at the start and end of each 20-min seawater and airflow halt cycle to determine the postprandial *M*DIC, *M*TAN and *M*urea-N. At 12, 24 and 48 h post-feeding, 1.5 L of seawater were collected and stored for the later determination of ^15^N ammonia enrichment^38,39^. After each measurement, the extracted water was replaced with freshly filtered seawater. After adding fresh seawater for the last time at 48 h, the lobster was euthanised in ice seawater slurry, weighed, measured, and dissected to remove the whole hepatopancreas (HP) and approximately 1 g WW of tail muscle tissue (TM). The carcass remains, HP and TM samples were individually stored at − 20 °C in preparation for freeze-drying and chemical composition analysis. Followed by the dissection, the ṀO_2_, *M*DIC, *M*TAN and *M*urea-N in the experimental vessel were measured 3 times for 20 min to identify differences in background parameters before and after each experiment.

#### Nitrogen excretion analysis

Nitrogenous concentrations were measured colourimetrically using a Synergy HT Multi-detection Microplate Reader (BioTek Instruments, Winooski, VT, USA). The concentration of *M*TAN was determined by the salicylate-hypochlorite method^40,41^. The concentration of *M*urea-N was determined with the diacetyl monoxime method^42^, which was modified to increase sensitivity^43^. The correlation coefficients (*R*^*2*^) of the linear calibration curves in *M*TAN and *M*urea-N determination were higher than 0.99. The duration (h) of *M*TAN, *M*urea-N and total nitrogenous (*M*TN, the sum of *M*TAN and *M*urea-N) excretion was determined when postprandial excretion rates returned to routine levels.

#### Nitrogen quotient and respiratory quotient calculation

The nitrogen quotient (NQ) was calculated as (*M*TN/14)/(ṀO_2_/32), and the respiratory quotient (RQ) was calculated as (*M*DIC/12)/(ṀO_2_/32), where 14, 32 and 12 are the atomic masses of N, O_2_, and C, respectively; *M*TN (μmol g^−1^ h^−1^) and *M*DIC (μmol g^−1^ h^−1^) represent the rate of total nitrogenous excretion (*M*TAN plus *M*urea-N) and total dissolved inorganic carbon excretion, respectively^17^.

#### Instantaneous metabolic energy substrate use calculation

The instantaneous metabolic energy substrate use was based on Wang, et al.^17^. The fraction of aerobic energy substrate use supplied by protein (amino acid) (P), lipid (L) and carbohydrate (C) was calculated as follows:

$$P = NQ/0.27$$

$$1.0 = P + L + C$$

$$RQ = \left( {m - 0.71} \right) \times NQ/0.27 + 0.29 \times C + 0.71$$

where 0.27 is the theoretical maximum nitrogen quotient (NQ) when protein (amino acid) is the only substrate being completely oxidised under aerobic conditions; m is the aerobic respiratory quotient (RQ) for protein (amino acid) oxidation, determined by 0.96 × *M*TAN% + 0.83 × *M*urea-N%, where 0.96 and 0.83 are the aerobic RQ for protein (amino acid) oxidation when ammonia and urea are the unique nitrogenous end-products, respectively; *M*TAN% and *M*urea-N% represent the contribution of *M*TAN and *M*urea-N to *M*TN (total nitrogenous excretion), respectively. The NQ was calculated as *M*TN/ṀO_2_ and RQ as *M*DIC/ṀO_2_, where *M*TN, ṀO_2_ and *M*DIC were expressed as µmol g DW^-1^ h^-1^ (“Nitrogen quotient and respiratory quotient calculation”).

#### Protein synthesis

Ammonia from the 1.5 L collected seawater was distilled into boric acid to form ammonium borate. Full details of ammonia distillation are given by Carter, et al.^39^ with modifications. The acidified sample is distilled with 40 anti-bump granules and a 30 mL mixture of 8 M NaOH and 0.1 M EDTA. Followed by distillation, ammonia is trapped as ammonium borate into 10 mL of 1 M boric acid. The ammonium borate was stored at − 20 °C and lyophilised (freeze-dried, FD). The FD ammonium borate samples were used to determine ^15^N enrichment (expressed as atom per cent excess, APE) of ammonia. Whole-body fractional protein synthesis rates (proportion of protein mass synthesised per day as a percentage, k_s_, % day^−1^) were calculated based on the ^15^N enrichment of ammonia, using the endpoint stochastic model in conjunction with the determined whole-body protein content (protein pool)^36,39^. Protein consumption rates (k_c_, % day^−1^) were calculated based on the final protein content of the lobster (g DW protein consumed g DW lobster protein^−1^ day^−1^, Houlihan, et al.^18^). Anabolic stimulation efficiency = 100 × k_s_/k_c_)^13^ provided further analysis of protein utilisation. Protein retention rates (k_g_) were calculated as the difference between digestible protein intake (dietary amino acid) and nitrogen excretion^44^. Protein degradation rates (k_d_) was calculated from k_g_ and k_s_ measurements following the equation k_d_ = k_s_ − k_g_^11^.

Two conversion factors were used to convert absolute protein synthesis rates into equivalent oxygen consumption values, the minimum theoretical cost of 46 mmol ATP g protein synthesis^−1^ and a determined energy cost of 69 mmol ATP g protein synthesis^−1^^20^, assuming that 5.24 mg O_2_ mmol ATP^−1^^13^.

### Chemical composition analyses

All samples, including experimental feeds, protein sources and animals, were freeze-dried (FD) to a constant weight and subsequently prepared for biochemical analysis by first grinding to a homogenous powder mechanically using an analytical mill (A11 basic Analytical mill, IKA®) and then manually using a mortar and pestle. Dry matter (DM) of FD samples was determined gravimetrically after oven drying at 105 °C for 24 h^45^. All biochemical analyses were performed on FD samples and corrected for DM. Ash content was determined by the combustion of FD samples in a furnace at 600 °C for 2 h^46^. Crude protein content was determined after measuring the elemental nitrogen (N) composition of FD samples using a flash combustion isotope ratio mass spectrometry (varioPYRO cube coupled to isoprime 100 mass spectrometer)^47^. A conversion factor of 6.25 × N was used to calculate crude protein. The total lipid content was determined gravimetrically using a modified method from Bligh and Dyer^48^. Briefly, total lipid was extracted in a mixture of dichloromethane, methanol and milliQ water (1:1:0.9 v/v/v) Yagiz, et al.^49^, except for the substitution of chloroform by dichloromethane^50^. Nitrogen free extract (NFE) was calculated as; NFE = 100 – (crude protein + total lipid + ash), g kg^−1^ DM basis. Energy content was calculated using factors 23.9 MJ kg^−1^, 39.8 MJ kg^−1^ and 17.6 MJ kg^−1^ for proteins, lipids and carbohydrates (NFE), respectively^27^.

### Statistical analyses

Mean values of the replicate aquarium (*n* = 3) in the digestibility experiment and the individual lobster (*n* = 5) for the protein synthesis determination are reported ± standard error of the mean (S.E.). All statistical analyses were performed using RStudio (Version 1.2.5042, © 2009–2020 RStudio, Inc.). Before analysis, all percentage data were arcsine-transformed. Data were tested for normal distribution and homogeneity using the Shapiro–Wilk and Bartlett's test, respectively. Apparent digestibility coefficient of the experimental feeds, survival, instantaneous metabolic energy use and WBPS were analised by an ANOVA followed by a Tukey's HSD post hoc test. Different Spirulina levels and the energetic cost of SDA were tested with the help of paird student’s t-test. A repeated measure of ANOVA was used to compare nitrogen quotient and respiratory quotient at different time points. All statistical tests were considered significant at *p* < 0.05. The magnitudes and duration of SDA were determined for each lobster individually (*n* = 5) by fitting fifth-degree (ṀO_2_ and *M*DIC) and sixth-degree (*M*TAN and *M*urea) polynomial regressions to the experimental data. Magnitudes were calculated from the integral between the regression line and the routine metabolism using the function *integrate()* in R. Durations were determined by calculating the intercepts of the regression lines with the routine metabolism.

## Results

### Apparent digestibility of Spirulina

Spirulina levels did not significantly affect lobsters' survival (ANOVA, F_2_ = 1.70, *p* = 0.259), the average survival rate was 67 ± 5%. The dry matter (DM), crude protein (CP), total lipid (TL), ash, nitrogen free extract (NFE), and gross energy (GE) in Spirulina were 91.6%, 70.0%, 4.7%, 9.6%, 14.9% and 21.3 MJ kg^−1^ dry matter, respectively. Ref and SP1 had significantly higher apparent digestibility coefficients (%) of DM (ADC_DM_), CP (ADC_CP_), TL (ADC_TL_) and GE (ADC_GE_) than SP2 (Table 2). The apparent digestibility of DM (AD_DM_), CP (AD_CP_) and GE (AD_GE_) were not significantly affected by Spirulina content (Table 3), and thus, an average nitrogen AD of 78 ± 3% was used for protein synthesis calculations.

**Table 2:** Apparent digestibility coefficients (ADC, %) for dry matter (ADC_DM_), crude protein (ADC_CP_), total lipid (ADC_TL_) and gross energy (ADC_GE_) in juvenile *Thenus australiensis* fed experimental feeds with different Spirulina content (mean ± S.E., n = 3).

	Experimental feeds			ANOVA	
	Ref	SP1	SP2	F	p
ADC_DM_	81.5 ± 0.6^b^	77.6 ± 1.3^b^	71.6 ± 1.5^a^	17.3	0.003
ADC_CP_	95.8 ± 0.3^b^	93.4 ± 0.9^b^	88.1 ± 0.7^a^	31.3	0.001
ADC_TL_	48.6 ± 3.4^b^	45.2 ± 0.9^b^	19.2 ± 3.3^a^	32.1	0.001
ADC_GE_	88.9 ± 0.1^b^	86.2 ± 0.6^b^	76.6 ± 1.1^a^	93.9	< 0.001

Significant differences (*p* < 0.05), determined by Tukey's HSD post hoc test, are denoted by superscript a to b.

**Table 3:** Apparent digestibility (AD, %) of dry matter (AD_DM_), crude protein (AD_CP_), and gross energy (AD_GE_) of Spirulina using 85:15 (SP1) and 70:30 (SP2) inclusion levels for juvenile *Thenus australiensis* (mean ± S.E., n = 3).

	Spirulina levels		*t* test	
	SP1 (15%)	SP2 (30%)	*t*	*p*
AD_DM_	54.3 ± 8.9	47.6 ± 5.3	1.46	0.281
AD_CP_	82.7 ± 5.1	73.7 ± 2.0	2.61	0.121
AD_GE_	68.4 ± 1.5	51.0 ± 3.1	3.86	0.061

### Oxygen consumption and specific dynamic action

**Figure 1:**
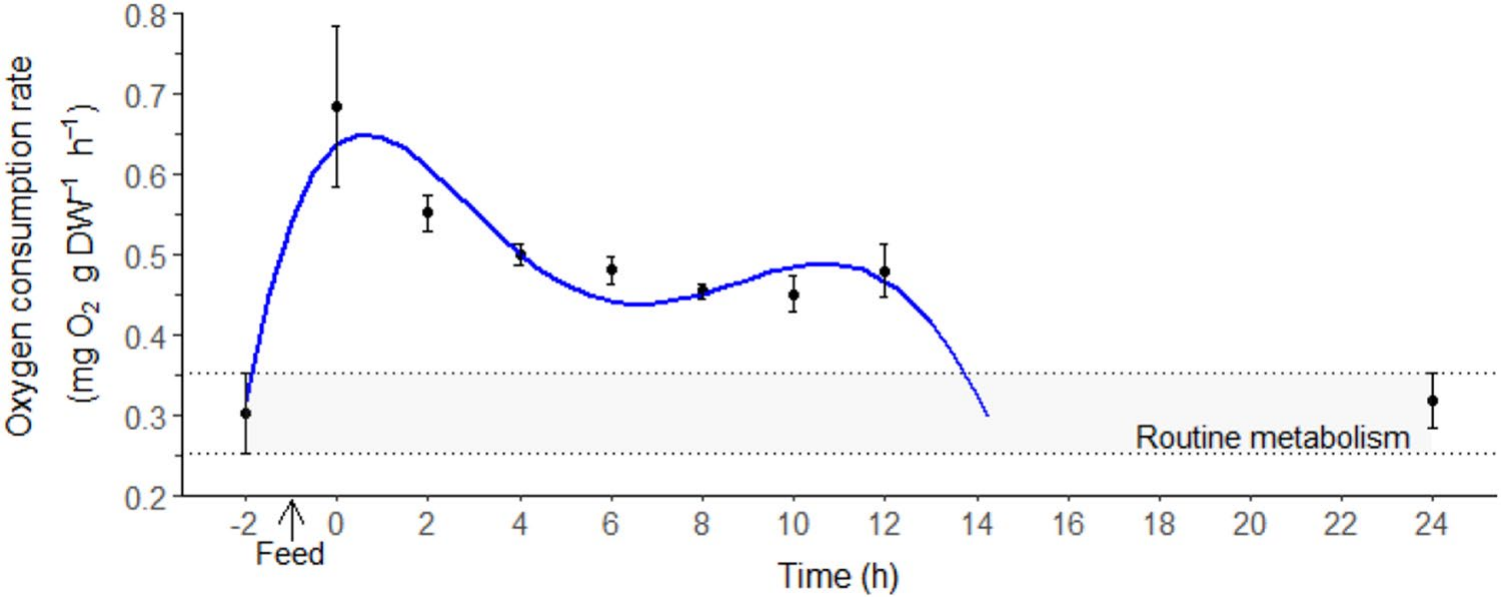
Oxygen consumption rate (mg O_2_ g DW^−1^ h^−1^) in juvenile *Thenus australiensis*. Lobsters were reared at 25.5 ± 0.3 °C and fed ^15^N-labeled feed. The ṀO_2_ at − 2 h indicates the routine metabolic rate (RMR), and 0 h was set as the first postprandial record. All data represent the mean ± standard error (S.E.) of five individuals. Blue line shows fifth-degree polynomial regression.

The oxygen consumption rates (ṀO_2_) of *T. australiensis* exhibited an immediate rise after feeding (0 h, Fig. 1) followed by a progressive drop to a RMR levels at 24 h post-feeding. Consequently, ṀO_2_ results were only reported for the first 24 h post-feeding. The specific dynamic action peak (SDA_peak_) of 0.68 ± 0.10 mg O_2_ g DW^−1^ h^−1^ was reached directly after the feeding period (0 h, Fig. 1). The SDA lasted on average 13 ± 2 h with a magnitude of 3.37 ± 0.78 mg O_2_ g DW^−1^. The energetic cost of SDA (E_SDA_) evaluated by two approaches did not differ significantly (*t* test, t_4_ = 2.93, *p* = 0.061) and were 46.6 ± 10.8 J g^−1^ (C_SDA_ = 2.3 ± 0.5%) and 32.8 ± 10.1 J g^−1^ (C_SDA_ = 1.6 ± 0.5%) for the simplified traditional and stoichiometric bioenergetic approaches, respectively.

### Nitrogen excretion and nitrogen quotient

**Figure 2:**
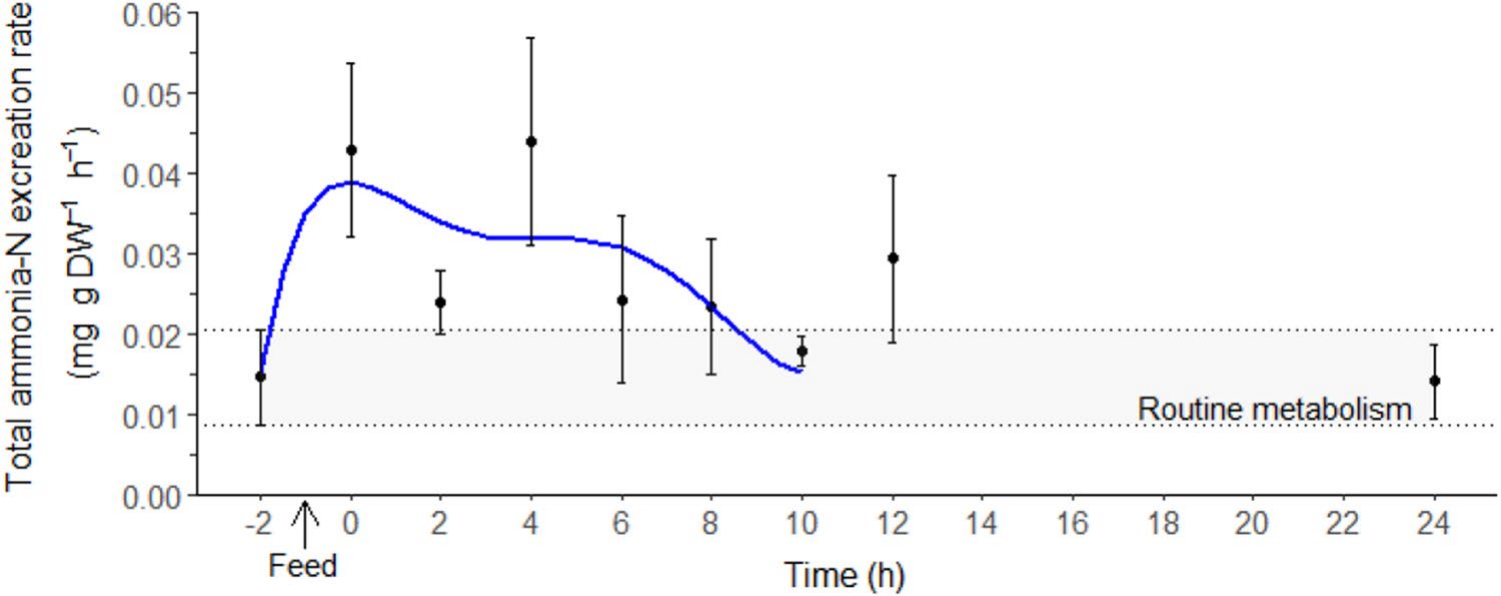
Total ammonia-N excretion rate (*M*TAN, mg g DW^−1^ h^−1^) in juvenile *Thenus australiensis*. Lobsters were reared at 25.5 ± 0.3 °C and fed ^15^N-labeled feed. The *M*TAN at − 2 h indicates the routine *M*TAN, and the first postprandial *M*TAN was recorded at 0 h. All data represent the mean ± standard error (S.E.) of five individuals. Blue line shows sixth-degree polynomial regression.

The routine total ammonia-N excretion of juvenile *T. australiensis* was 0.015 ± 0.006 mg g DW^−1^ h^−1^ (− 2 h, Fig. 2). Ammonia excretion rates increased rapidly after feeding and an *M*TAN peak of 0.044 ± 0.010 mg g DW^−1^ h^−1^ was reached after 4 ± 1 h. The *M*TAN duration lasted on average 4 ± 1 h with a magnitude of 0.727 ± 0.272 mg g DW^−1^.

**Figure 3:**
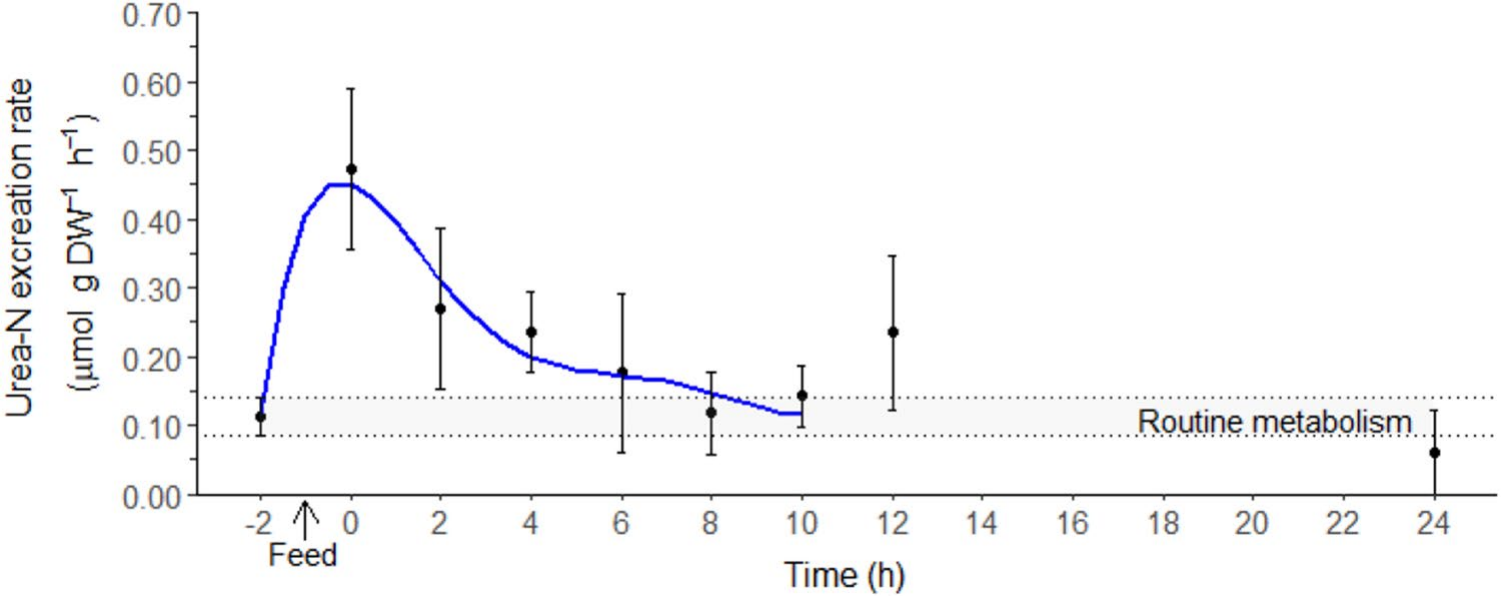
Urea-N excretion rate (*M*urea-N, µmol g DW^−1^ h^−1^) in juvenile *Thenus australiensis*. Lobsters were reared at 25.5 ± 0.3 °C and fed ^15^N-labeled feed. The *M*urea-N at − 2 h indicates the routine *M*urea-N, and the first postprandial *M*urea-N was recorded at 0 h. All data represent the mean ± standard error (S.E.) of five individuals. Blue line shows sixth-degree polynomial regression.

The routine *M*urea-N excretion of juvenile *T. australiensis* was 0.11 ± 0.03 µmol g DW^−1^ h^−1^ (− 2 h, Fig. 3). *The M*urea-N peak of 0.52 ± 0.04 µmol g DW^−1^ h^−1^ was reached directly after the feeding period (− 2 h, Fig. 3). The *M*urea-N duration lasted 5 ± 1 h with a magnitude of 2.07 ± 0.43 µmol g DW^−1^.

**Figure 4:**
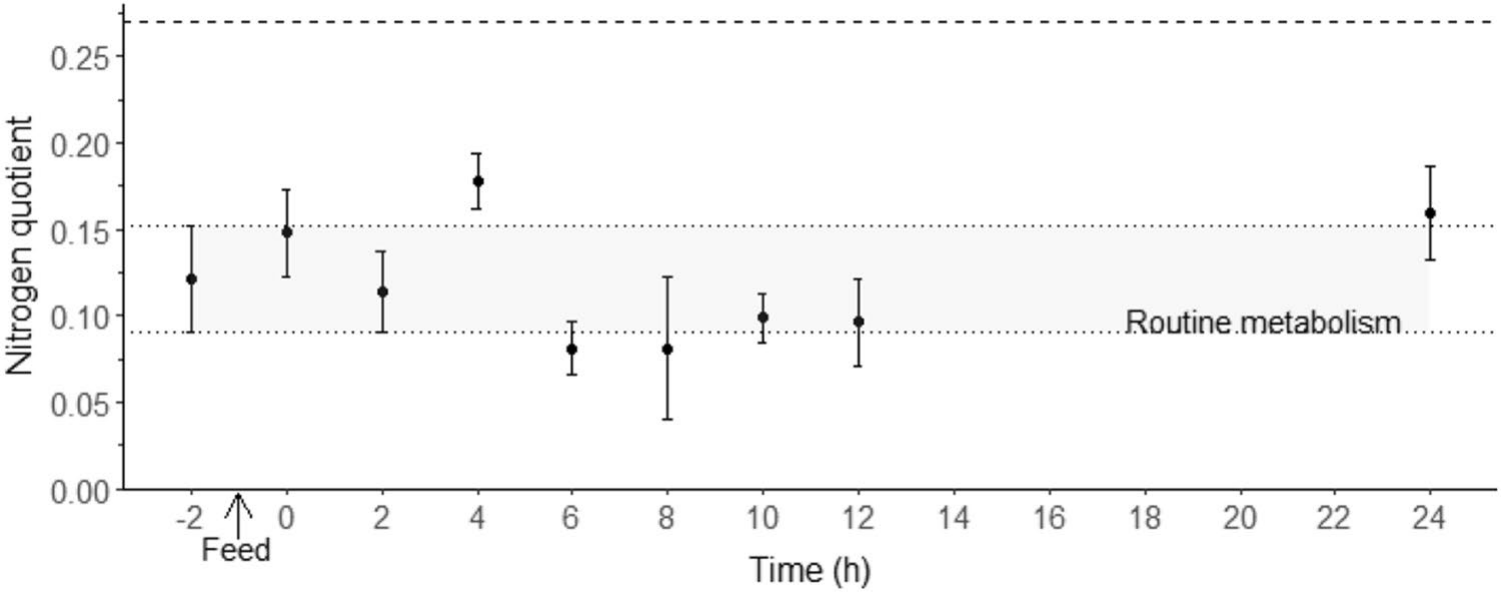
Nitrogen quotient (NQ) in juvenile *Thenus australiensis*. Lobsters were reared at 25.5 ± 0.3 °C and fed ^15^N-labeled feed. The NQ at − 2 h indicates the routine NQ, and the first postprandial NQ was recorded at 0 h. The dashed line indicates the theoretical maximum nitrogen quotient of 0.27. All data represent the mean ± standard error (S.E.) of five individuals.

Nitrogen quotient was not significantly different at the different time points (ANOVA, F_8, 32_ = 0.71, *p* = 0.684, Fig. 4).

### Total dissolved inorganic carbon excretion and respiratory quotient

**Figure 5:**
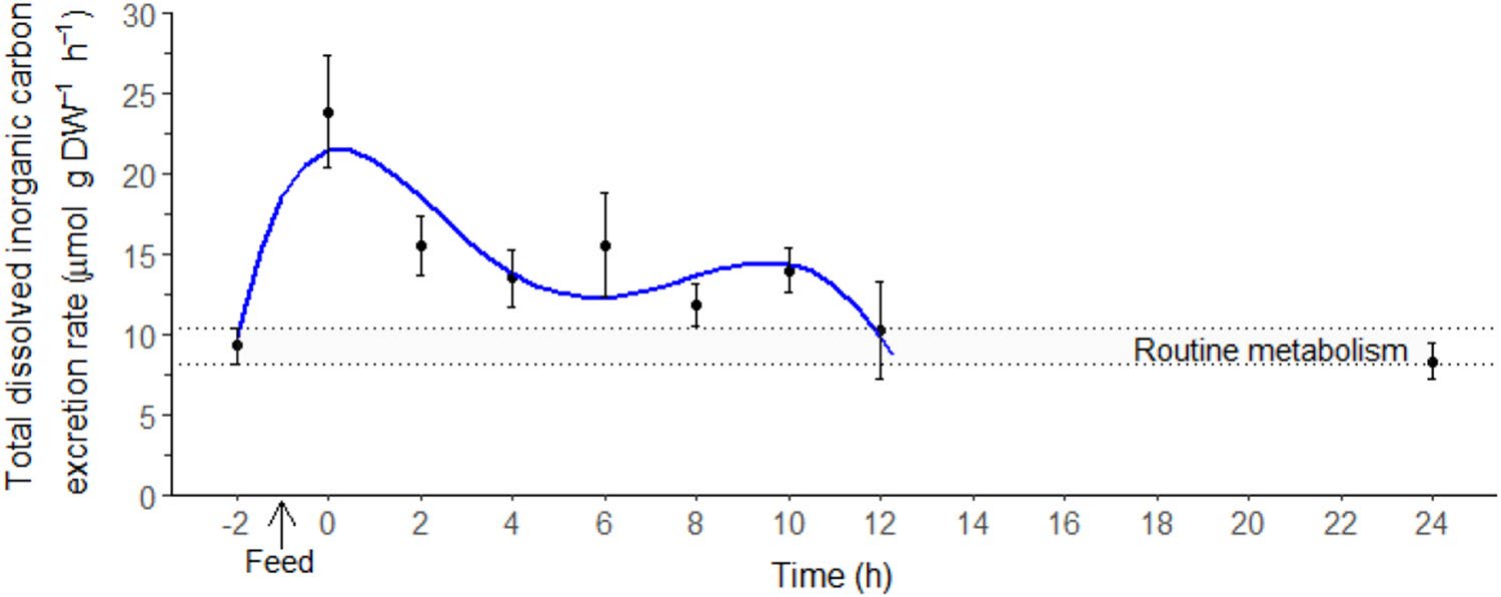
Total dissolved inorganic carbon excretion rate (*M*DIC, µmol g DW^−1^ h^−1^) in juvenile *Thenus australiensis*. Lobsters were reared at 25.5 ± 0.3 °C and fed ^15^N-labeled feed. The *M*DIC at − 2 h indicates the routine *M*DIC, and the first postprandial *M*DIC was recorded at 0 h. All data represent the mean ± standard error (S.E.) of five individuals. Blue line shows fifth-degree polynomial regression.

The routine dissolved inorganic carbon excretion rate (*M*DIC) was 9.28 ± 1.12 µmol g DW^−1^ h^−1^ (− 2 h, Fig. 5). Juvenile *T. australiensis* showed an immediate postprandial increase in *M*DIC, presenting a peak of 24.7 ± 3.1 µmol g DW^−1^ h^−1^ directly after the feeding period (0 h, Fig. 5). The *M*DIC duration lasted 9 ± 1 h with a magnitude of 84.1 ± 22.4 µmol g DW^−1^.

**Figure 6:**
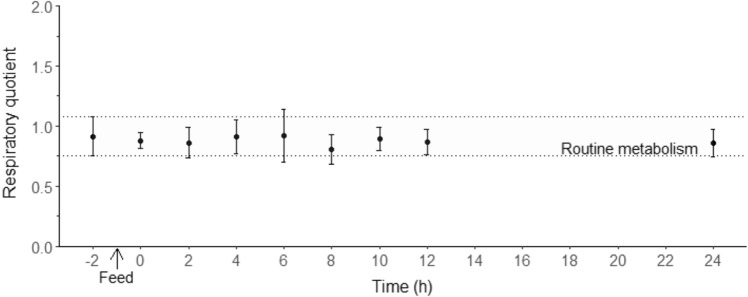
Respiratory quotient (RQ) in juvenile *Thenus australiensis*. Lobsters were reared at 25.5 ± 0.3 °C and fed ^15^N-labeled feed. The RQ at − 2 h indicates the routine RQ, and the first postprandial RQ was recorded at 0 h. All data represent the mean ± standard error (S.E.) of five individuals.

Respiratory quotient did not differ significantly between different time points (ANOVA, F_8, 24_ = 0.75, *p* = 0.649, Fig. 6).

### Instantaneous metabolic energy use

**Figure 7:**
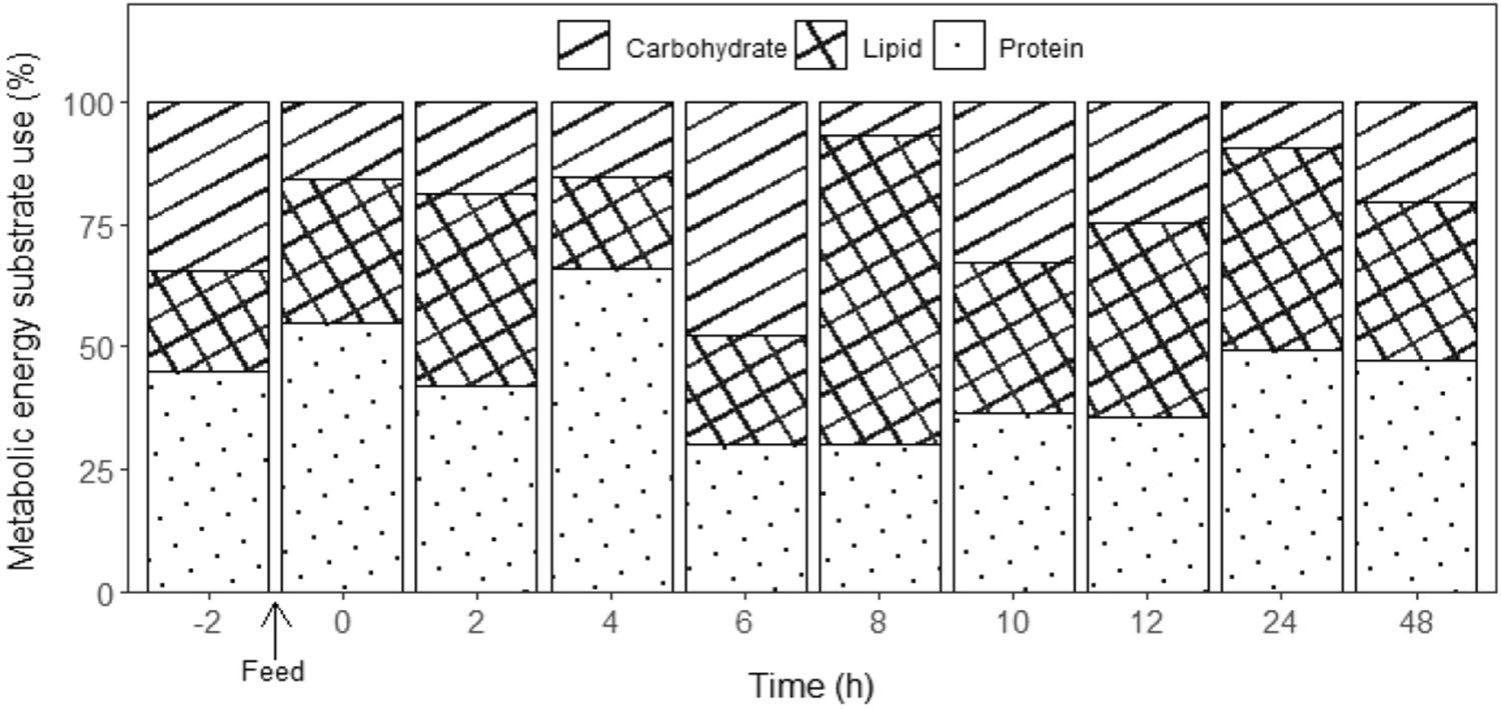
Instantaneous metabolic energy substrate use (%) in juvenile *Thenus australiensis*. Lobsters were reared at 25.5 ± 0.3 °C and fed ^15^N-labeled feed. The percentage at − 2 h indicates the metabolic energy substrate use during routine metabolism. The first postprandial metabolic energy substrate use was recorded at 0 h. All data represent the mean values of five individuals.

Protein (amino acid, 45%) was the predominant metabolic energy substrate in 48 h fasted lobster, followed by carbohydrate (35%) and lipid (20%, Fig. 7). The fractional contribution to metabolic use of protein (amino acid), carbohydrate and lipid fluctuated during the 48 h post-feeding, totalling 44%, 21% and 35%, respectively. The pairwise comparison revealed that protein (amino acid) had a significantly higher percentage of metabolic energy substrate use than carbohydrates (Tukey's HSD, *p* = 0.002), with no significant differences found between protein (amino acid) and carbohydrate with lipid (*p* > 0.05).

### Protein synthesis

**Figure 8:**
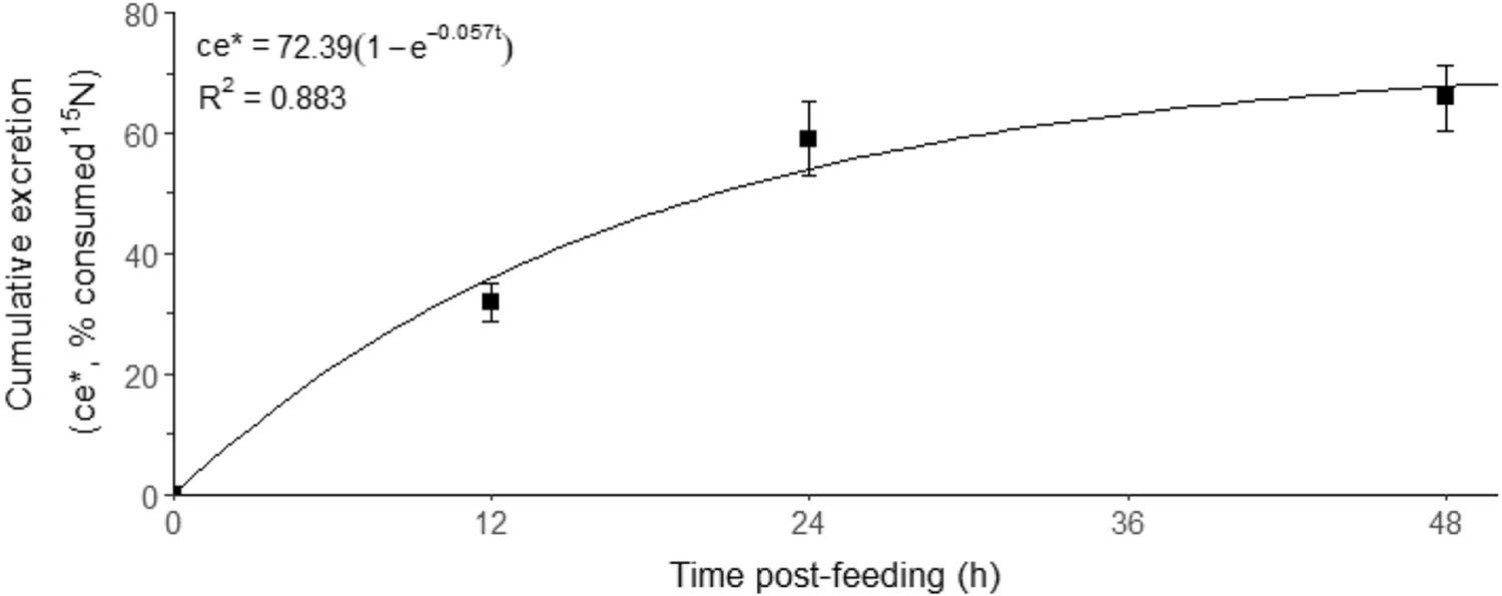
Cumulative ^15^N-labeled ammonia excretion rate (ce*, %, expressed as the percentage of ^15^N in the assimilated feed) in juvenile *Thenus australiensis* (*n* = 5) over 48 h post-feeding. Lobsters were reared at 25.5 ± 0.3 °C and fed ^15^N-labeled feed. Data points represent the mean ± standard error (S.E.) of five individuals and four sampling times.

The cumulative ^15^N-labeled ammonia excretion rate (ce*) was measured over 48 h following a single feed (Fig. 8). The relationship between ce* and time after feeding was described as a negative exponential regression, showing a plateau between 24 and 48 h post-feeding. Thus, WBPS was estimated for 24 and 48 h following feeding using data collected over the first 48 h post-feeding.

**Figure 9:**
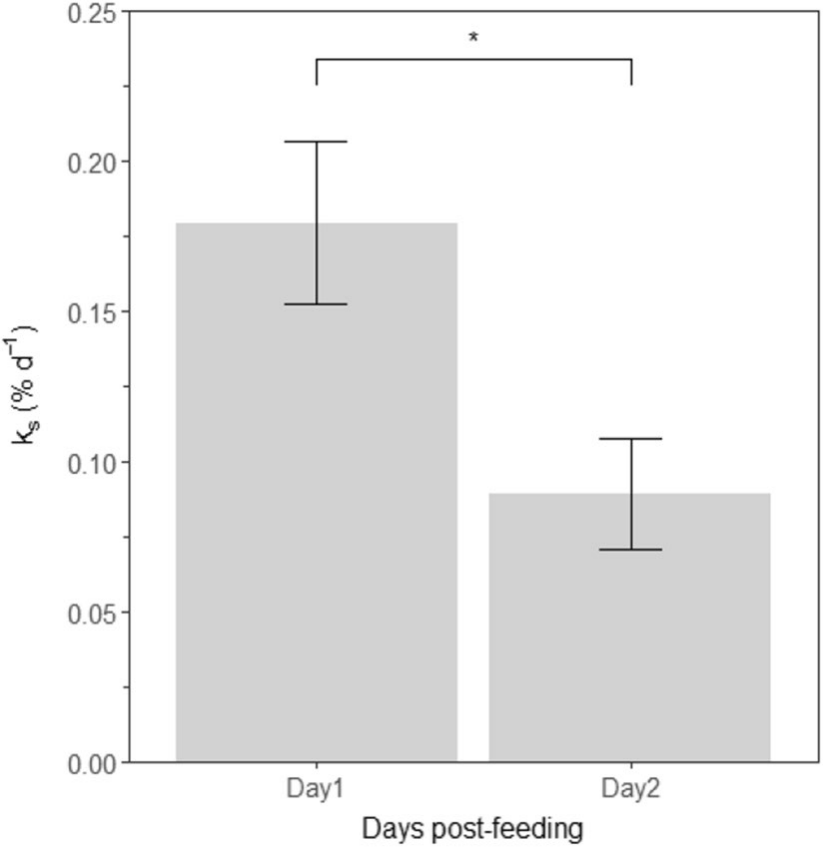
Fractional whole-body protein synthesis rates (k_s_% day^−1^) in juvenile *Thenus australiensis* estimated over 48 h post-feeding. Lobsters were reared at 25.5 ± 0.3 °C and fed ^15^N-labeled feed. Data represent the mean ± S.E. of five individuals, *p* < 0.05.

The whole-body protein content (protein pool) of juvenile *T. australiensis* was 503 ± 30 g CP kg^−1^. The WBPS and fractional protein synthesis (Fig. 9 and Table 4) were determined to be 0.76 ± 0.15 mg CP g^−1^ day^−1^ and 0.15 ± 0.03% day^−1^, respectively. Protein synthesis on the second day was significantly reduced (ANOVA, F_1_ = 8.47, *p* = 0.020, Fig. 9) compared to day 1. The postprandial increase in WBPS accounted for 13 ± 3% and 19 ± 5% of total oxygen uptake, using the minimal and experimentally determined cost of protein synthesis in ATP g^−1^ protein, respectively.

**Table 4:** Protein turnover and efficiency parameters for juvenile *Thenus australiensis* at 25.5 ± 0.3 °C (*n* = 5).

	Day 1	Daily average
k_c_ (% day^−1^)	5.75 ± 0.68	2.88 ± 0.34
k_a_ (% day^−1^)	4.47 ± 0.10	2.23 ± 0.27
k_a_/k_c_ (%)	77.66 ± 3.28	77.66 ± 3.28
k_s_ (% day^−1^)	0.21 ± 0.04	0.15 ± 0.03
WBPS (mg CP g^-1^ day^−1^)	1.07 ± 0.21	0.76 ± 0.15
ASE (%)	3.80 ± 0.67	3.65 ± 0.99

*k*_*c*_ protein intake, and *k*_*a*_ digestible protein intake, *k*_*s*_ protein synthesis, *ASE* Anabolic stimulation efficiency (k_s_/k_c_).

## Discussion

The present study validates the successful combination of a stoichiometric bioenergetic approach with an endpoint stochastic model for the simultaneous determination of SDA, metabolic substrate use and WBPS in aquatic ectotherms first used by Wang, et al.^6^. A comprehensive understanding of the contribution of protein synthesis to metabolism is key to investigating the relationship between nutritional status and feed nutritional quality and the effects on growth potential in crustaceans^13,15^. Thus, the present study provides comprehensive data on the major components of whole-animal metabolism, including respiratory gas exchange, nitrogenous excretion, specific dynamic action, metabolic energy substrate use, and whole-body protein synthesis in juvenile slipper lobster *Thenus australiensis*. Each of these components must be assessed individually and validated against previous research before they can be considered collectively to describe the protein-nitrogen flux revealing the nutritional status of the animal.

The in vivo method for digestibility determination is subjected to different factors affecting its accuracy^51^. In juvenile white shrimp *Litopenaeus vannamei* apparent digestibility of test ingredients were significantly affected by the ingredient type and inclusion level, except for AD_CP_^51^. In the present study, spirulina levels resulted in significantly different apparent digestibility coefficients for the experimental feeds. However, Spirulina digestibility was not affected by different inclusion levels, and AD_CP_ equalled 78 ± 3%. The AD_CP_ for Spirulina, in the present study, was higher than previous reports of 52.6% in *Macrobrachium tenellum*^52^ and 52.9% in *S. verreauxi*^6^. Since the same Spirulina protein was used for *S. verreauxi*^6^ and *T. australiensis*, the different protein digestibilities may result from differences in the digestive capacity of these species, emphasising the importance of determining digestibility when protein synthesis is determined by ^15^N-labeled nitrogen^36^.

SDA is a well-known metabolic event^16,53,54^, and following a meal, the flux of amino acids and other nutrients from the digestive system into other tissues stimulates a rapid increase in metabolism reflected by peaks in oxygen consumption, nitrogen excretion and protein synthesis^53,55^. Hence, postprandial oxygen consumption increases above the pre-feeding rate, often to a peak level, and then falls back to the pre-feeding level^54^. In the present study, oxygen consumption of *T. australiensis* agrees and showed a 2.3-fold increase in ṀO_2_ directly after the meal, followed by a decline back to RMR within 24 h.

The energetic cost of SDA (E_SDA_) estimated by two different approaches (one simplified traditional approach and one stoichiometric bioenergetic approach) were not significantly different and in agreement with previous findings for *S*. *verreauxi*^6^. However, since the ESDA values determined in the present study showed a difference of 14% (47 − 33%), further research is needed to understand if the traditional approach, using an empirical oxycalorific coefficient to convert SDA magnitude to energy, provides accurate E_SDA_ results. The E_SDA_ in the present study was notably higher than for other aquatic ectotherms^6,31^. At a ration of 1.5% BW, E_SDA_ ranged from 3.0 J g^−1^ in kelp crab (*Pugettia producta*) to 10.9 J g^−1^ in purple shore crabs (*Hemigrapsus nudus*), and with increasing E_SDA_ with increasing feed ration^31^. The SDA magnitude and, consequently, E_SDA_ in aquatic ectotherms depends on body weight, feed composition, feeding ration, and temperature^16,31,54,56^. Hence, the SDA coefficient (C_SDA_), calculated by dividing E_SDA_ by the dietary energy content^54,57^, allows for intraspecific and interspecific comparisons of SDA that are independent of meal size, meal type, body size, and body temperature^31,53^. Further, the combination of C_SDA_ and nitrogenous excretion provides information about dietary protein utilisation. Whereas optimal dietary protein can result in lower SDA and nitrogenous excretion^58^, an increase in C_SDA_ and nitrogen excretion indicates increased protein metabolism and decreased efficiency in converting ingested nutrients to growth^6,56^. In the present study, the low C_SDA_ of 2.31% and 1.62%, simplified traditional and stoichiometric bioenergetic approach, respectively, are comparable with previously reported C_SDA_ for *S. verreauxi* (1.8–2.6%)^6^, indicating efficient use of the provided formulated feeds.

As protein synthesis is a major contributor to SDA, it typically responds in a similar way, increasing after feeding before gradually returning to pre-feeding levels^15,16,20^. The present study results agree and show a parallel postprandial increase of ṀO_2_ and k_s_, a return to RMR and a significant decrease of k_s_ after 24 h. A significant proportion of SDA can be accounted for by the elevation of protein synthesis because SDA largely represents the cost of protein anabolism and hence growth^59^. The cost of WBPS in *T. australiensis* accounted for 13–19% (26 °C) of total oxygen uptake, agreeing with previous findings in crustaceans and fish^13,20^.

Ammonia-N excretion in juvenile *T. australiensis* was comparable with other decapod crustaceans^30,60^ and represented the primary nitrogenous end-product with 83% of the total nitrogenous excretion. The *M*urea-N measured in the present study equalled 17% of the total nitrogenous excretion, similar to previous reports for *Orconectes rusticus*^61^.

Investigating the instantaneous metabolic substrate use provides a deeper understanding of how aquatic ectotherms oxidise energy substrates to provide energy and evaluate the dietary protein-sparing effect^17,23^. In the present study, the primary energy substrate oxidised in 48 h fasted lobster was protein (amino acid), followed by carbohydrate and lipid agreeing with other short-term fasted carnivorous decapods findings^17,62,63^. Provided protein (amino acid), carbohydrate and lipid were all oxidised at different proportions at different times to provide energy over 48 h post-feeding, indicating that besides protein, appropriate proportions of non-protein energy substrates, lipid and carbohydrate, are both likely to be essential for the formulation of cost-effective aquafeeds^17,64^.

Protein synthesis is central to growth; consequently, the study of WBPS is key to understanding the daily protein-nitrogen flux in aquatic ectotherms, which is crucial for optimising dietary protein in aquafeeds^6,24,25^. The present study used an endpoint stochastic model to calculate protein synthesis rates, previously applied to *S. verreauxi*^6^. Successful application of the single endpoint model depends on the ^15^N-labeled nitrogen appearing in a single major nitrogenous end-product^36,37^. In the present study, ammonia was the major nitrogenous end-product with low *M*urea-N excretion. Consequently, using ^15^N-labeled ammonia to calculate WBPS is suitable for *T. australiensis*. The validation data on cumulative ^15^N-labeled ammonia excretion rates showed a plateau within 48 h post-feeding, agreeing with findings for other aquatic ectotherms^65,66^.

Information on postprandial WBPS in crustaceans is limited^6,20,22,67^. Whilst in *G. antarcticus* (0 °C, 33 g) and isopod *Saduria entomon* (1 g, 4 and 13 °C) fed at 5% BW k_s_ equalled 0.4% day^−1^, 1.5% day^−1^ (4 °C) and 2.6% day^−1^ (13 °C), at the SDA_peak_, respectively^22,67^. Feeding (3% BW) *C. maenas* (15–18 °C, 54 g) showed a k_s_ of 2.6% day^−1^^20^. The differences in postprandial k_s_ can be explained by the combination of factors, including water temperatures, body weights, feeding regimes, methods and the different species used^15,67,68^. Using the same methodology, feeding regime, and dietary protein content of 60%, *S. verreauxi* (21 °C, 993 ± 23 g) showed a similar k_s_ of 0.19% day^−1^^6^ to the k_s_ reported in the present study (0.15 ± 0.03% day^−1^, 26 °C, 9.6 ± 0.3 g). However, since protein turnover is affected by BW and protein synthesis and protein degradation decline with increasing BW^11,69^, *T. australiensis*, in the present study, should have presented higher protein synthesis rates than *S. verreauxi*. A major driver of metabolism is the nutritional status of the animal, governed by feeding, nutrient intake and quality, and time without food^15,33^. Thus, low k_s_ may be due to the poor nutritional status of the lobsters, resulting in low feed intake, which was lower than previously reported for juvenile *T. australiensis*^8^.

**Figure 10:**
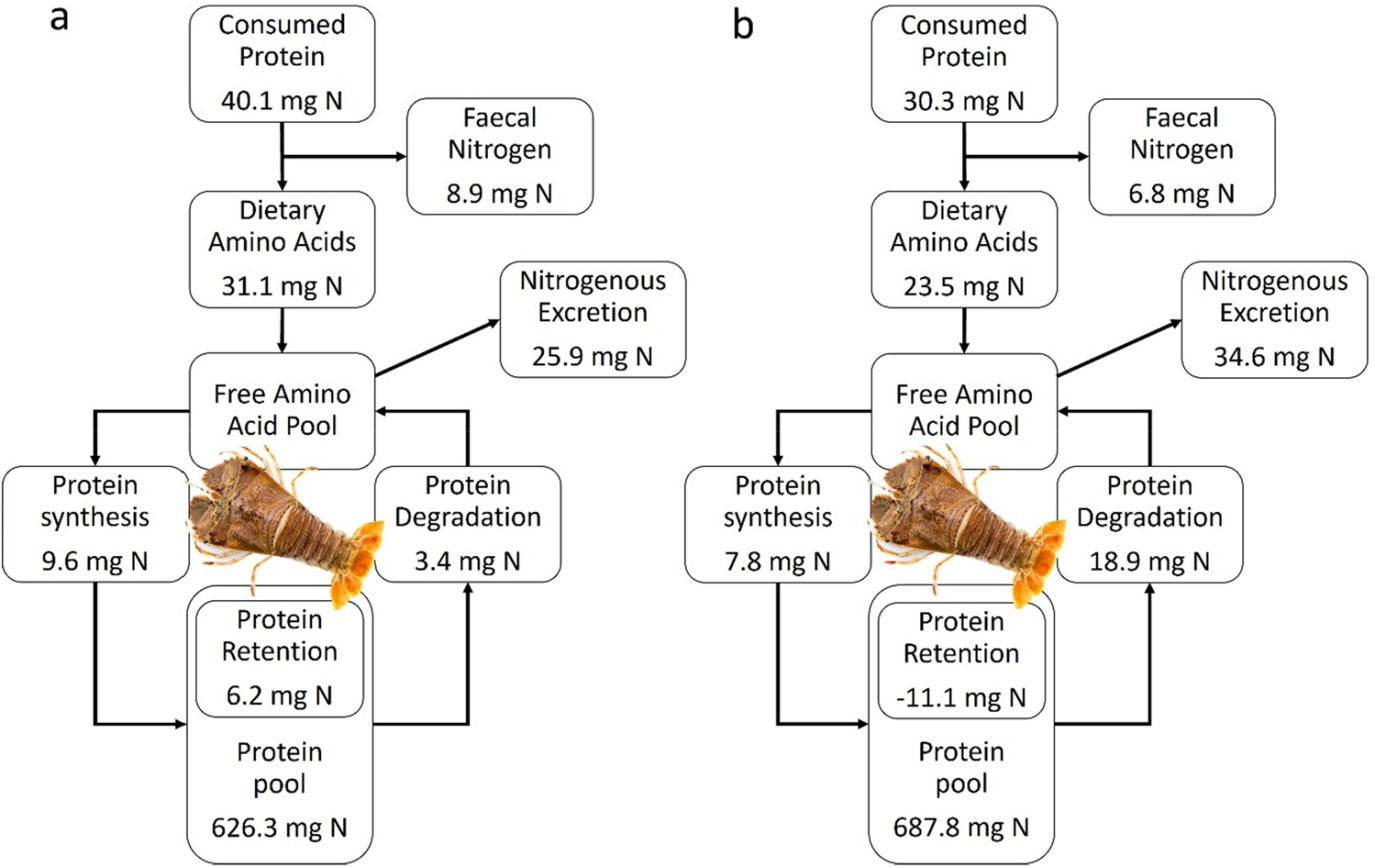
Protein-Nitrogen flux (mg N day^−1^) for a theoretical 50 g WW juvenile *Thenus australiensis* for the first 24 h following the consumption of a single meal showing a: protein growth (positive protein retention) and b: protein loss (negative protein retention) based on Carter, et al.^44^.

The comprehensive investigation on the major components of whole-animal metabolism contributes to our understanding of nutritional status and feed nutritional quality in relation to WBPS. Measuring protein synthesis and protein-nitrogen flux provides a subtle approach to assessing the efficiency of dietary protein utilisation^13^, thereby expanding knowledge of nutritional physiology and energy metabolism. Protein-nitrogen flux describes the partitioning of consumed and absorbed protein-nitrogen into protein syntheses and growth. In a simple nitrogen budget, the daily protein-nitrogen flux derived from consumed protein partitioned between faecal waste (faecal nitrogen), metabolic waste (total nitrogen excretion), and growth (protein retention)^13,70,71^. Fractional protein synthesis rates from the present study (Table 4) were used to model the protein turnover rates of a theoretical 50 g WW juvenile *T. australiensis* calculated for the first 24 h following consumption of a single meal based on Carter, et al.^44^. The protein-nitrogen flux was separately calculated for lobster with a positive (*n* = 2, Fig. 10a) and negative protein retention (*n* = 3, Fig. 10b). Increasing feed intake and corresponding digestible protein intake (dietary amino acids) increased protein synthesis and protein retention, agreeing with previous findings for the grass carp (*Ctenopharyngodon idella*)^44^. Since all lobsters were fed the same experimental feed, the protein loss can be explained by the poor nutritional status of the lobsters, displayed by low protein consumption and protein synthesis^15,33^. Thus, modelling protein-nitrogen flux is a helpful tool to gain deeper insight into nutritional physiology, specifically understanding the partitioning of consumed and absorbed protein-nitrogen in juvenile *T. australiensis*.

## Conclusion

The present study successfully combined a stoichiometric bioenergetic approach with an endpoint stochastic model for the simultaneous determination of SDA, metabolic substrate use and WBPS in juvenile *T. australiensis*. The combined approach has great potential to provide crucial nutritional and metabolic information about the nutritional status of *T. australiensis*. The low k_s_ determined in the present study can be linked to the poor nutritional status of the animal governed by low feed intake. A balanced feed containing suitable protein and other non-protein energy substrates such as lipid and carbohydrate is essential to spare dietary protein and increase protein efficiency for growth. In the present study, protein was the primary energy substrate, suggesting that dietary protein was not efficiently used for growth. Consequently, more research is required to investigate the effect of dietary protein on routine and postprandial nutritional physiology and bioenergetics. Thus, future research should consider integrating protein synthesis into protein requirement experiments of marine ectotherms to acquire a more comprehensive picture of nutritional requirements and efficiency.

## Data Availability

Data presented in this study are available on request from the corresponding author.
